# Acute appendicitis induced by bone fragment ingestion: A pediatrics case report

**DOI:** 10.1016/j.radcr.2024.08.010

**Published:** 2024-08-27

**Authors:** Nguyen Xuan Khai, Nguyen Viet Dung, Truong Dinh Tien, Dao Minh Hai, Le Dang Thanh Cong, Nguyen Ngoc Khanh, Tran Van Duy, Do Thanh Nam, Ngo Tuan Minh

**Affiliations:** a Department of Interventional Radiology, Radiology Center, 103 Military Hospital, Vietnam Military Medical University, Hanoi, Vietnam; b Department of Radiology, 108 Military Central Hospital, Hanoi, Vietnam; c Department of Pathology, 103 Military Hospital, Vietnam Military Medical University, Hanoi, Vietnam; d Paraclinical Department, Tan Dan Clinic, Bacgiang, Vietnam; e Department of Radiology and Endoscopy, Phu Quoc Medical Center, Kiengiang, Vietnam; f Class YKTHK4, Thanh Hoa Campus, Hanoi Medical University, Hanoi, Vietnam; g Department of Pediatrics, 103 Military Hospital, Vietnam Military Medical University, Hanoi, Vietnam; h Department of Gastroenterology and Hepatology, 103 Military Hospital, Vietnam Military Medical University, Hanoi, Vietnam

**Keywords:** Bone fragment, Foreign body, Acute appendicitis, Computed tomography

## Abstract

Acute appendicitis is an ordinary surgical emergency, typically attributed to luminal obstruction by fecaliths or lymphoid hyperplasia. However, ingested foreign bodies as an etiology are rare but increasingly recognized, particularly in pediatric patients. We present the case of a 9-year-old male patient who presented to the emergency department with symptoms consistent with acute appendicitis. Further investigation revealed the presence of a bone fragment within the appendix, leading to acute inflammation. Foreign body ingestion should be considered in pediatric patients with acute appendicitis. This case report underscores the importance of comprehensive clinical evaluation and appropriate diagnostic imaging modalities in guiding optimal treatment strategies.

## Introduction

Appendicitis is one of the most common surgical emergencies, with an incidence rate of 233 per 100,000 population, accounting for 300,000 hospitalizations annually in the United States [1]. The symptoms of the condition typically lack specificity, manifesting as abdominal pain near the umbilicus that migrates to the lower right abdomen, accompanied by fever, nausea, and vomiting. The cause of appendicitis is believed to be obstruction of the appendix lumen, caused by lymphoid hyperplasia, fecaliths, parasitic infections, tumors, and quite rare, foreign body ingestion [2].

Foreign body ingestions occur in over 100,000 individuals annually in the United States. Among them, over 80% are children, and 98% of these cases result from accidental ingestion [3]. Typically, ingested foreign bodies may pass through the digestive system without causing symptoms. However, due to the higher prevalence of foreign body ingestions in children, coupled with narrower gastrointestinal tracts compared to adults, they are more prone to developing complications. The incidence rate of acute appendicitis due to foreign bodies is approximately 0.0005% [4]. Among the commonly ingested objects, rigid and sharp items have a higher likelihood of entering the appendix and causing inflammation, possibly leading to appendiceal perforation.

Here, we report a 9-year-old male case of acute appendicitis caused by ingesting an animal bone fragment, which was successfully treated with laparoscopic appendectomy surgery. This report details the clinical approach in practice and the computed tomography (CT) imaging features associated with this rare condition.

## Case presentation

A male patient at 9 years old presented with continuous right iliac fossa abdominal pain for 2 days before admission. On the day of admission, the patient's colicky pain intensified, accompanied by fever and chills, without associated anorexia, nausea, or vomiting. He was admitted to the hospital for examination and treatment.

On examination, the patient exhibited diffuse abdominal tenderness, predominantly localized in the right iliac fossa region, with positive guarding and rebound tenderness. McBurney's point tenderness was positive. The patient had a fever of 37.9°C. Blood tests revealed a white blood cell count of 6.8 × 10^9/L, with a slightly elevated percentage of neutrophils at 70.3% (4.8 × 10^9/L). According to the Pediatric Appendicitis Score (PAS) (Table 1) [5], the patient scored 5 points, showing an indeterminate risk of appendicitis. However, abdominal ultrasound did not reveal any abnormalities. The patient was scheduled for noncontrast-enhanced CT imaging of the abdomen to diagnose the underlying cause of abdominal pain.

**Table 1:** Pediatric appendicitis score.

Nausea/vomiting	1
Anorexia	1
Migration of pain to RLQ	1
Fever	1
Cough/percussion/hopping tenderness	2
RLQ tenderness	2
Leucocytosis (WBC > 10,000)	1
Neutrophilia (ANC > 7500)	1
Low risk: PAS ≤ 3; Indeterminate risk: PAS 4-6; High risk: PAS ≥ 7	

Source: M. Samuel (2002), "Pediatric appendicitis score". J Pediatr Surg, 37(6), 877–81 [5].

**Fig. 1:**
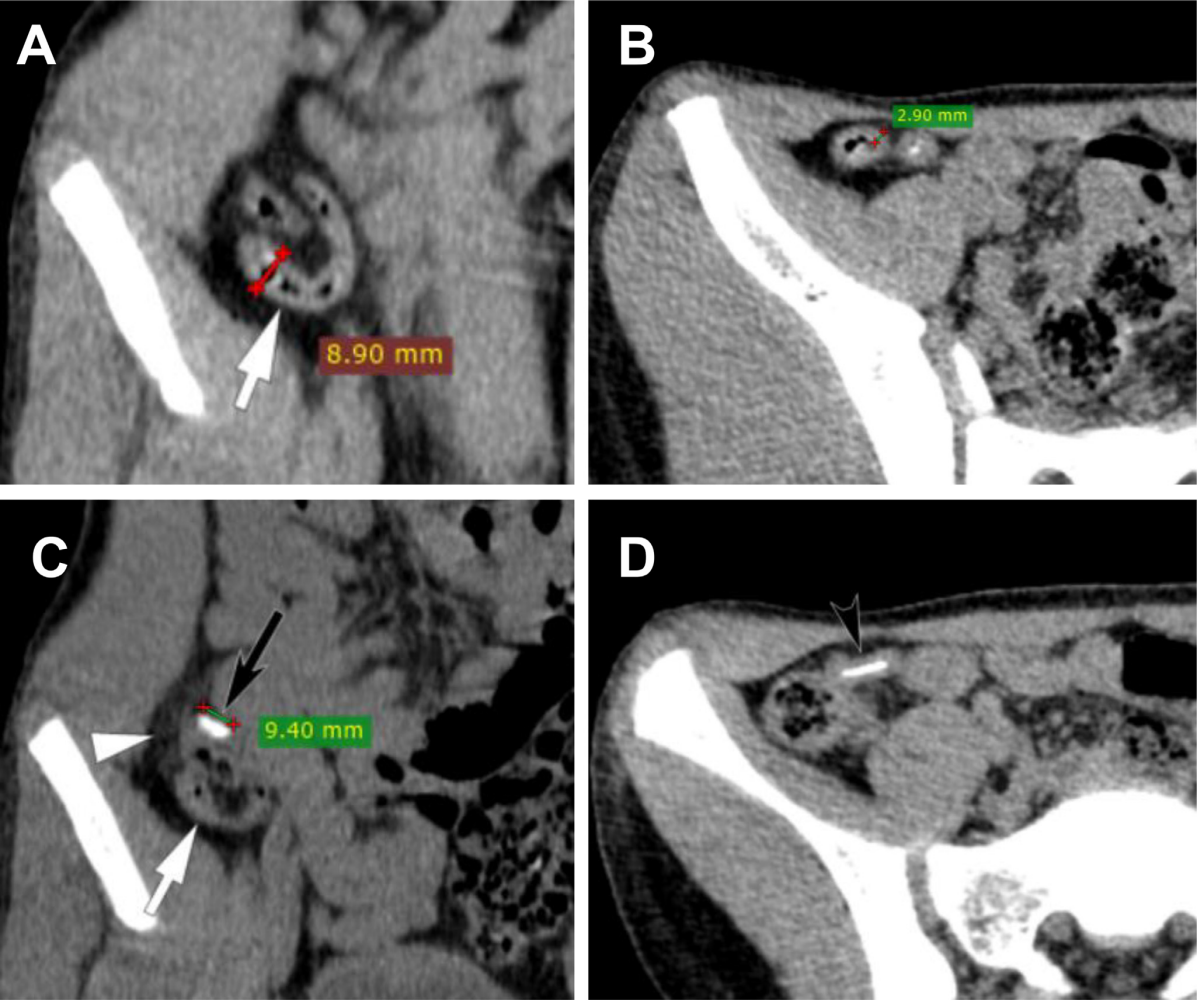
The abdominopelvic computed tomography images: (A, B) the appendix appeared enlarged, with a diameter of 8.9 mm and a wall thickness of 2.9 mm. (C) A radiopaque foreign body (black arrow), approximately 9.4 mm in length with well-defined edges, within the lumen of the appendix (white arrow). Axial CT image (C) also shows subtle fat stranding around the base of the appendix near a foreign object (white arrow head), in contrast to the body of the appendix, which shows no infiltration (black arrow head in image D).

The appendix appeared enlarged on the noncontrast-enhanced CT images, with a diameter of 8.9 mm and a wall thickness of 2.9 mm. There was mild peri-appendiceal inflammation. A radiopaque foreign body, with a length of approximately 9.4 mm and well-defined edges, was noted at the base of the appendix. The CT imaging suggested a foreign body at the appendix base, suspected to cause acute appendicitis (Fig. 1).

**Fig. 2:**
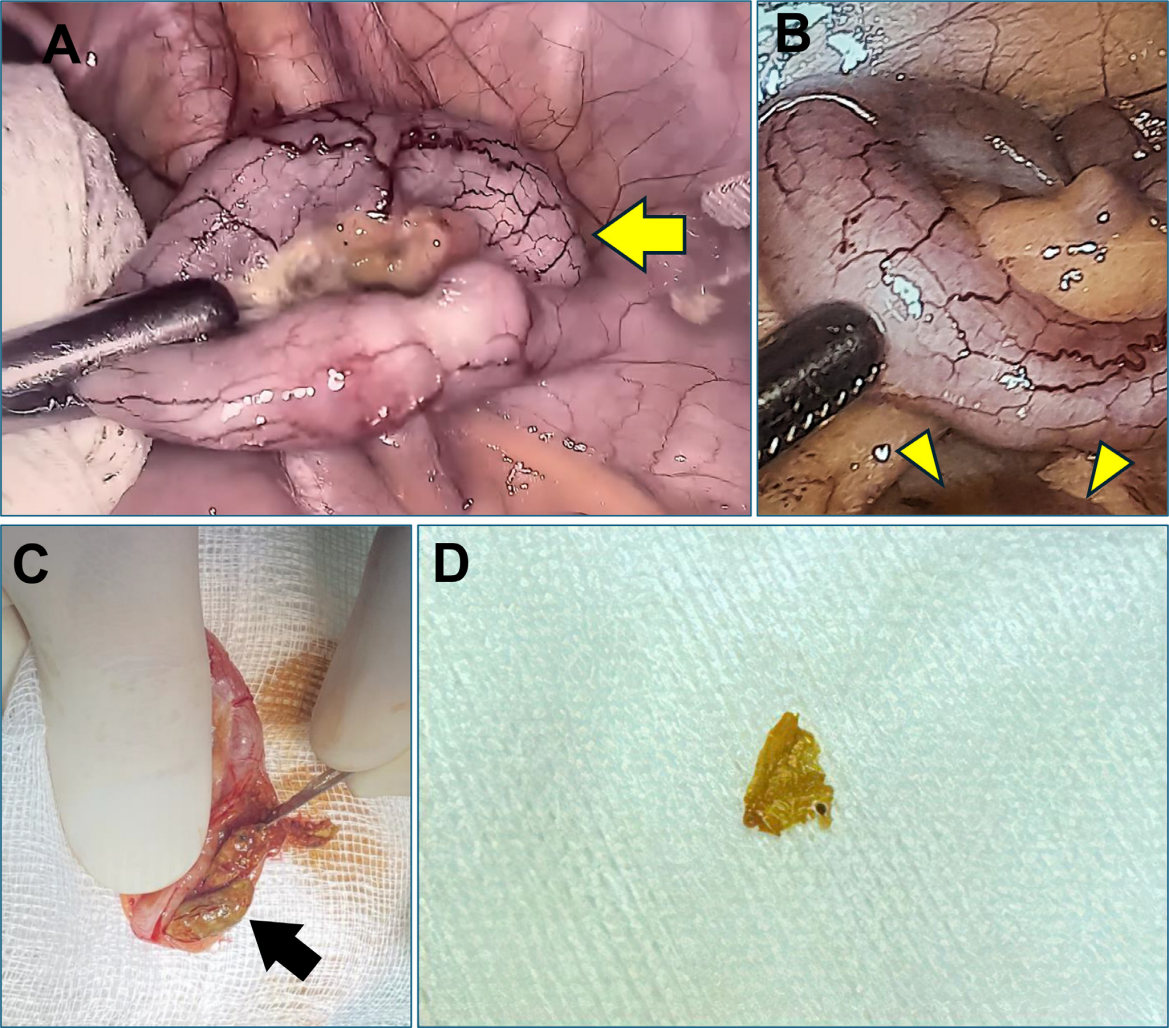
(A) Intraoperative laparoscopic images showing the appendix appears swollen (arrow) with hyperemia of serosal vessels and adjacent visceral peritoneum, (B) Exudate found in the right Iliac foss (arrowhead). (C, D) The resected appendix contains a bone fragment within the lumen (black arrow).

**Fig. 3:**
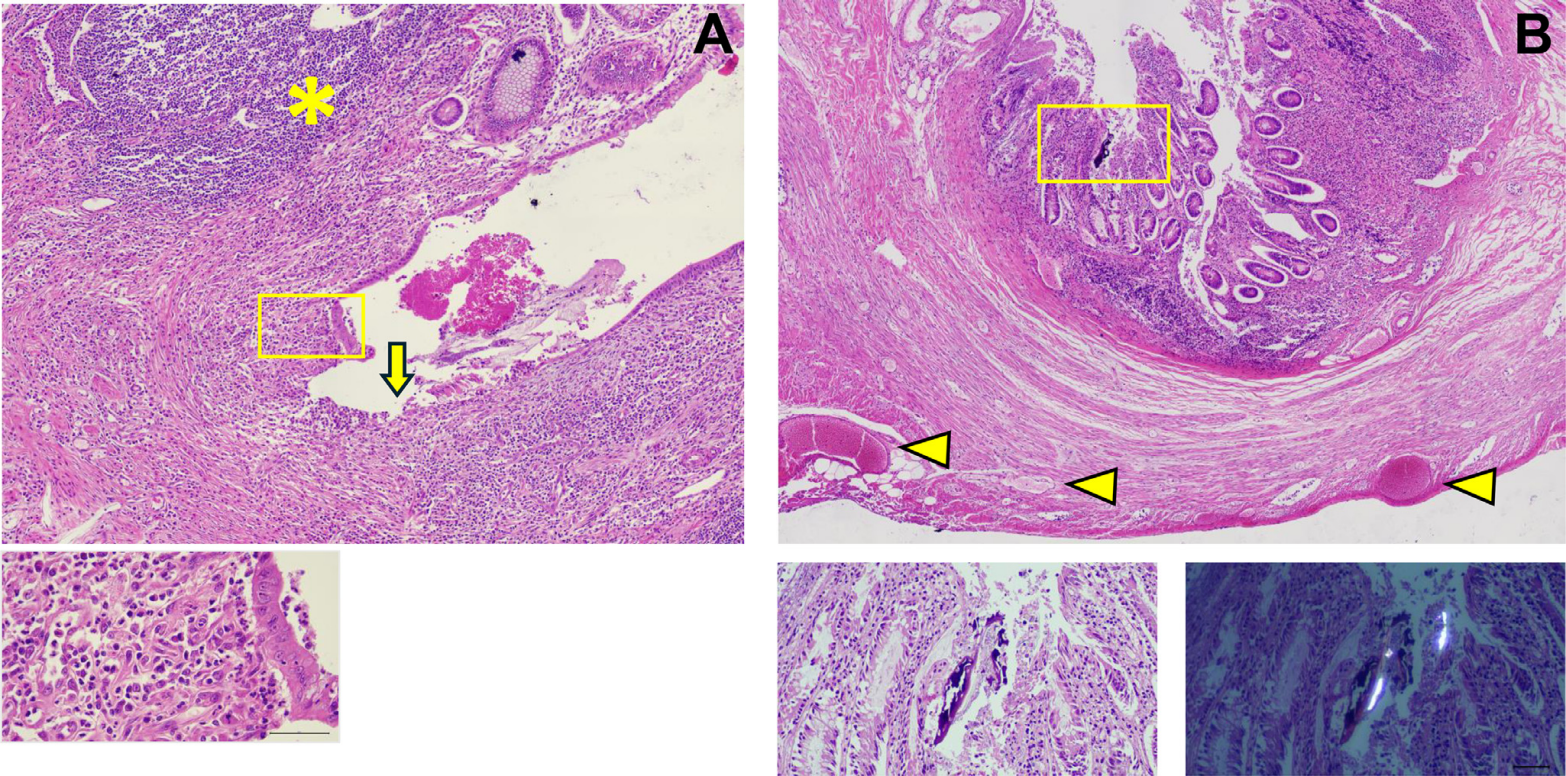
(A) Microscopic appearance of the affected appendix reveals enlargement of the lymphoid follicle (*), mucosal erosion (arrow), and neutrophil infiltration into the lamina propria (inset) (H&E, x200); (B) The bone fragment was found within the mucosa (inset), partly enhancing contrast under polarized light; The appendix wall showed exudate with extensive vessels congested (arrowhead) (H&E, x100).

During the surgical procedure, free fluid was observed localized in the right iliac fossa, and the appendix appeared swollen and congested. The surgeon proceeded with an appendectomy, revealing a fragment of animal bone within the appendix lumen (Fig. 2). The histopathological study showed the early stage of acute appendicitis with the bone fragment in the mucosa layer (Fig. 3). Postoperative recovery progressed smoothly, and the patient was discharged 6 days after surgery. The combination of the intraoperative laparoscopic view and the pathological study supported the diagnosis of acute appendicitis related to a foreign body.

## Discussion

### Pathogenesis

Foreign body ingestion is a common concern, particularly in pediatric patients, involving objects unexpectedly swallowed and lodged in the gastrointestinal (GI) tract. Children, exclusively usual in infants and toddlers, are prone to ingesting foreign bodies due to their natural curiosity and tendency to explore objects orally. Additionally, young children may not have fully evolved fine motor skills or understanding of potential dangers, leading them to swallow objects without notice while playing or eating. Moreover, some children may swallow objects because of developmental disorders, behavioral issues, or other medical conditions. Most foreign bodies can pass through the digestive tract without symptoms or complications. However, in less than 10% of cases, foreign bodies may become struck in the esophagus, stomach, or another part of the GI tract, leading to complications such as perforation, intestinal obstruction, or local inflammation [6]. The incidence of acute appendicitis due to foreign body ingestion is very low, approximately 0.0005% [4]. According to the literature, common types of ingested foreign bodies include screws, needles, bone fragments, dentures, and pins, with sharp objects having a higher complication rate. Several hypotheses suggest that the weight of the foreign body causes it to become trapped in the appendix, which lies at the base of the cecum. Once inside the appendix, the peristalsis is not strong enough to push the foreign body back into the colon [7]. The foreign body may stimulate an inflammatory reaction, leading to inflammation and perforation of the appendix wall in the case of sharp objects, or it may remain asymptomatic for an extended period. Therefore, the time from ingestion to the appearance of appendicitis symptoms can range from a few hours to several years [2].

### Clinical symptoms and diagnosis

The PAS is utilized to assess the risk of appendicitis in children. This scoring system relies on clinical factors such as abdominal pain, vomiting, fever, and laboratory parameters like white blood cell count [8,9]. Based on the score, patients can be categorized into different risk groups. In cases of nonspecific symptoms of appendicitis in children, utilizing the PAS helps determine risk and guide treatment. In our patient, some typical symptoms of acute appendicitis were present, including fever (37.9°C), abdominal pain initially in the epigastric region, then migrating to the right lower quadrant, positive McBurney's point tenderness, and guarding tenderness [10]. The patient scored 5 points, indicating an indeterminate risk of appendicitis.

When the PAS score indicates moderate risk, potential options include seeking a surgical consultant, diagnostic imaging, and serial abdominal examinations while under hospital observation, depending upon local resources. Ultrasonography and CT are the modalities used most frequently. Ultrasonography can identify appendicitis; however, it may be challenging to differentiate between appendicitis caused by fecaliths or foreign bodies. CT imaging can detect signs of appendicitis, including thickening of the appendix wall, surrounding inflammation, or the presence of a foreign body. In cases where there is suspicion of foreign body ingestion, particularly bone fragments or other objects, CT scanning proves instrumental in precisely localizing and characterizing the object within the gastrointestinal tract. CT imaging provides detailed anatomical information on surrounding structures, enabling clinicians to anticipate potential complications, such as appendiceal abscess, perforation, or peri-appendiceal inflammation [6]. This diagnostic clearness helps physicians make therapeutic decisions and subsequent disease management protocols. In our case, abdominal ultrasound did not detect the foreign body within the appendix; hence, we needed to order a CT scan to confirm the diagnosis.

### Management

The treatment of ingested foreign bodies aims to minimize potential complications. In case of acute appendicitis, open or laparoscopic appendectomy is necessary to remove the foreign body and reduce complications [11]. According to the guidelines of the European Association for Endoscopic Surgery (EAES), laparoscopic appendectomy is considered the preferred approach for all cases of acute appendicitis [12]. Open surgery may be utilized but is associated with prolonged hospital stays and may increase the likelihood of negative appendectomies [13]. In our case, the patient underwent laparoscopic appendectomy, which resulted in a successful outcome, with the patient making a full recovery.

## Conclusion

Although rare, ingesting foreign bodies can be a cause of appendicitis, especially in children. Therefore, physicians should pay attention in clinical practice by thoroughly exploring the patient's medical history and utilizing various imaging diagnostic methods.

CT scanning not only aids in accurately diagnosing the foreign body as the cause of appendicitis but also allows for the assessment of severity and any accompanying complications. Removal of the foreign body is a crucial part of treatment. CT scans offer detailed images of the location and shape of the foreign body, enabling surgical teams to identify its position and extent before proceeding with surgery. CT is essential in making treatment decisions, especially in cases where ultrasound findings are inconclusive.

## Data availability

The data supporting this article are available from the authors on reasonable request.

## Author contribution

Conceptualization, Nguyen Xuan Khai, Dao Minh Hai, and Ngo Tuan Minh; Writing - original draft, Nguyen Xuan Khai, Nguyen Viet Dung, and Ngo Tuan Minh. Undergoing the diagnostic procedure, collecting, and interpreting the imaging and pathology, Nguyen Xuan Khai, Nguyen Viet Dung, Le Dang Thanh Cong, Dao Minh Hai, Truong Dinh Tien, Tran Van Duy, Nguyen Ngoc Khanh, Do Thanh Nam, and Ngo Tuan Minh. Writing, review & editing, Nguyen Xuan Khai, Nguyen Viet Dung, Truong Dinh Tien, and Ngo Tuan Minh. All authors have read, revised, and agreed to the final published version of the manuscript.

## Patient consent

We have obtained written informed consent from the patient's parent to publish this case report. The patient's parent consented to de-identified clinical information and images being used for this report. The authors of the manuscript retain this informed consent and can provide it to the journal upon request.
